# Prediction Model of Anastomotic Leakage Among Esophageal Cancer Patients After Receiving an Esophagectomy: Machine Learning Approach

**DOI:** 10.2196/27110

**Published:** 2021-07-27

**Authors:** Ziran Zhao, Xi Cheng, Xiao Sun, Shanrui Ma, Hao Feng, Liang Zhao

**Affiliations:** 1 Thoracic Surgery Department National Cancer Center/National Clinical Research Center for Cancer/Cancer Hospital Chinese Academy of Medical Sciences and Peking Union Medical College Beijing China; 2 Department of Global Health Management School of Public Health and Tropical Medicine Tulane University New Orleans, LA United States; 3 Department of Epidemiology School of Public Health and Tropical Medicine Tulane University New Orleans, LA United States

**Keywords:** anastomotic leakage, esophageal cancer, esophagectomy, machine learning, risk factors

## Abstract

**Background:**

Anastomotic leakage (AL) is one of the severe postoperative adverse events (5%-30%), and it is related to increased medical costs in cancer patients who undergo esophagectomies. Machine learning (ML) methods show good performance at predicting risk for AL. However, AL risk prediction based on ML models among the Chinese population is unavailable.

**Objective:**

This study uses ML techniques to develop and validate a risk prediction model to screen patients with emerging AL risk factors.

**Methods:**

Analyses were performed using medical records from 710 patients who underwent esophagectomies at the National Clinical Research Center for Cancer between January 2010 and May 2015. We randomly split (9:1) the data set into a training data set of 639 patients and a testing data set of 71 patients using a computer algorithm. We assessed multiple classification tools to create a multivariate risk prediction model. Our ML algorithms contained decision tree, random forest, naive Bayes, and logistic regression with least absolute shrinkage and selection operator. The optimal AL prediction model was selected based on model evaluation metrics.

**Results:**

The final risk panel included 36 independent risk features. Of those, 10 features were significantly identified by the logistic model, including aortic calcification (OR 2.77, 95% CI 1.32-5.81), celiac trunk calcification (OR 2.79, 95% CI 1.20-6.48), forced expiratory volume 1% (OR 0.51, 95% CI 0.30-0.89); TLco (OR 0.56, 95% CI 0.27-1.18), peripheral vascular disease (OR 4.97, 95% CI 1.44-17.07), laparoscope (OR 3.92, 95% CI 1.23-12.51), postoperative length of hospital stay (OR 1.17, 95% CI 1.13-1.21), vascular permeability activity (OR 0.46, 95% CI 0.14-1.48), and fat liquefaction of incisions (OR 4.36, 95% CI 1.86-10.21). Logistic regression with least absolute shrinkage and selection operator offered the highest prediction quality with an area under the receiver operator characteristic of 72% in the training data set. The testing model also achieved similar high performance.

**Conclusions:**

Our model offered a prediction of AL with high accuracy, assisting in AL prevention and treatment. A personalized ML prediction model with a purely data-driven selection of features is feasible and effective in predicting AL in patients who underwent esophagectomy.

## Introduction

Esophagectomies are important treatments for early-stage and locoregionally advanced esophageal cancer. However, esophagectomies are burdened with a high incidence of complications. Anastomotic leakage (AL), including cervical anastomotic leakage and intrathoracic anastomotic leakage, is a significant complication following an esophagectomy accounting for morbidity and mortality (5%-30%) [1]. Moreover, it is associated with prolonged intensive care unit stays, reduced quality of life, and higher hospital costs [2]. Accordingly, the prevention and optimal management of AL after an esophagectomy are of great importance. Investigations should be undertaken as soon as the risk factors of AL are recognized because any delay would substantially worsen the prognosis [3]. The timely detection of surgical and nonsurgical AL risk factors and the adoption of a proper approach are keys to the successful treatment of AL.

Previous conventional prediction models exploring AL risk factors have not validated their model’s performance and provided the rationale for feature selection in their work. Analyses of several known predictive factors of AL have yielded poor statistical performance across studies [4]. Therefore, no consistent and clear predictive factors can be used to target patients with a risk of AL in clinical practice. Machine learning (ML) approaches are particularly suited to predictions based on real world evidence, which involves a computer algorithm learning important features of a data set and capturing complex relationships in the data to enable predictions about other unseen data. ML ensures a more accurate and robust prediction than conventional statistical models since it can capture nonlinear relationships among clinical features without human-biased intervention. It can predict AL for individual patients more accurately in terms of model performance and generalizability [5,6].

This study aims to use ML techniques to explore the risk factors that influence the occurrence of AL and the consequent clinical outcomes after an esophagectomy to inform the clinical management of AL. Various medical strategies are available to prevent AL after an esophagectomy, including patient screening and preparation, technical-surgical details, and postsurgical care management. Thus, the evidence generated from the prediction model can serve as a practical guide to the clinical management of patients undergoing esophagectomies with a particular focus on AL prevention. 

## Methods

### Study Design and Participants

In this retrospective study, we collected data on 710 patients who underwent an esophagectomy for esophageal cancer at the Department of Thoracic Surgery in the National Clinical Research Center for Cancer in China between January 2010 and May 2015. Our data were collected by manual chart review. The protocols and guidelines for data abstraction from the medical record were developed prior to launching the medical record data collection effort in our hospital to ensure the reliability and accuracy of the data collection. Our medical data investigator was trained carefully and followed strict protocols of data collection. Any discrepancies were reviewed jointly and discussed with our medical team to clarify any issues. The Independent Ethics Committee had approved this retrospective cohort study of the institution, and the requirement to obtain informed consent was waived.

We collected 76 features from patients’ medical records, including patient-related information such as demography (age, gender, and BMI), smoking and alcohol consumption, surgical history, prescriptive medication, and the American Society of Anesthesiologists (ASA) classification. The comorbidities registered included diabetes mellitus, malnutrition, hypertension, other cardiovascular diseases (cardiac arrhythmia and coronary heart disease), chronic obstructive pulmonary disease (COPD), etc. Intra-operative features included timing of surgery, type of operation, incisions, and blood transfusions. The data set identified two kinds of esophagectomy, including open esophagectomy and minimally invasive esophagectomy (MIE). Besides total MIE, thoracoscopic or laparotomy assisted esophagectomy, or hybrid MIE were also included. The three most common techniques for thoracic esophageal cancer were: (1) the transhiatal approach, (2) Ivor Lewis esophagectomy, and (3) the McKeown technique. The postoperative features included hospital stay, complications, and mortality, etc.

### Outcome Definition

The primary outcome of this study is a diagnosis of AL which was ascertained through clinical symptoms and confirmed by endoscopy, radiological examination, clinical examination of the anastomosis, or reoperation [7]. The Esophagectomy Complications Consensus Group defined AL as a “full-thickness gastrointestinal defect involving the esophagus, anastomosis, staple line, or conduit irrespective of presentation or method of identification” [8]. In our clinical practice, an AL is first suspected if there were any (1) clinical signs, such as fever, abdominal pain, feculent drainage, purulent drainage, or signs of peritonism; (2) radiographic signs, such as fluid collection or gas containing collection; and (3) signs of anastomotic dehiscence during endoscopy. The definitive diagnostic tool for suspected AL is a computerized tomography scan with a contrast of the abdomen and pelvis, which will demonstrate the presence of any extraluminal contents. An additional assessment is urgent blood tests, including full blood count, a coagulation screen, etc [9].

### Data Management and Machine Learning Approaches

**Figure 1:**
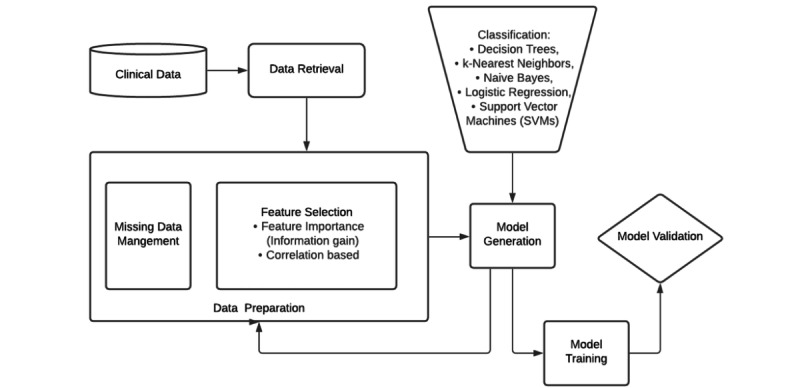
Analysis workflow for data management and model development.

Figure 1 illustrates the construction of the ML model and how the risk predictors (features) were handled. We first checked missing conditions and the balance of the data set; no variables were reported to have a missing percentage over 5%, meaning the completeness rate of every variable was above 95%. For this study, we randomly split the large data set (9:1) into a training data set (n=639) and a testing data set (n=71) using a computer algorithm. We split the data set using this ratio to allow sufficient training data to quantify the model’s complexity while maintaining adequate data to validate the model [10]. The cross-validation process was iterated 9 times. Model parameters were optimized by a grid search, greedily tuning the model hyperparameters. The mean area under the receiver operating characteristic curve (AUROC) was used to determine which model performed best and further tested with testing data set. The sensitivity level of AUROC is set to 90%, which is considered clinically relevant.

#### Identification of Risk Factors (Feature Selection)

Identifying the most important features was based on the two most used feature selection filter methods in ML: (1) feature importance and (2) correlation-based feature selection. We used filter methods of feature selection because it is independent of the potential models [11]. Feature importance is a univariate filter that compares each feature’s correlation with the outcome separately and removes features with zero importance according to a gradient boosting machine (GBM) learning model. Generally, importance provides a score indicating how useful or valuable each feature is in constructing the boosted decision trees within the model. The feature importance is averaged over 10 training runs of the GBM to reduce variance [12,13]. The correlation-based method is a multivariate filter that identifies the collinear features and removes the redundant features that are highly correlated with one another. These 2 feature selection methods have advantages in that they are more stable than the traditional statistical approaches, such as backward logistic regression, and they considerably minimized the model’s over-fitting problem [13]. Similar to previous medical ML studies, we performed the 2 feature selection methods on all 76 features using our training data set and initially identified N features that have the least correlation to AL, then plotted the change in AUROC for the prediction of AL from 1 to N features [13]. The ML algorithm also plotted the change in cumulative importance and recognized the least number of features required, receiving above 99% of the cumulative importance. Thus, we included the smallest number of independent features into the final prediction model.

#### Model Generation, Training, and Validation

Once our features were defined, we considered five different ML classification models (classifiers) to build our models: (1) logistic regression with regularizations, (2) a support vector machine using Gaussian kernel, (3) a decision tree based on information gain, (4) a random forest including 9 decision tree classifiers based on Gin impurity, and (5) a naive Bayes classifier assuming a Gaussian distribution. These 5 algorithms were chosen for comparison because they are well-accepted ML methods typically used in medical applications [10]. Finally, models were validated with our testing data set, and the extended metrics (AUROC, accuracy, recall, F1 score, and precision) were reported.

### Statistical Analysis

This study aligns with TRIPOD (Transparent Reporting of a Multivariable Prediction Model for Individual Prognosis or Diagnosis) and TRIPOD-ML guidelines (see Multimedia Appendices 1 and 2) [14,15]. The complete set of patient medical data was utilized to maximize the power and generalizability of our results. All the analyses were performed using sklearn, pandas, numpy, and lightgbm packages in Python (version 3.6.1).

In the descriptive summary, categorical variables were presented as numbers and percentages and continuous variables as mean and standard deviation. The *P* values were also provided for the association of each factor with the presence and absence of AL using the Pearson chi-square method. The ranked risk features panel from the training data set provided the importance and regression coefficients of the association of each feature in the final prediction model. Finally, we presented the risk factors of AL associated with each feature using odds ratios (OR) and 95% CI.

### Ethics Approval and Consent to Participate

This retrospective cohort study was approved by the institutional review board of the National Cancer Center/National Clinical Research Center for Cancer/Cancer Hospital’s Ethics Committee, and the requirement to obtain informed consent was waived.

## Results

Demographic and symptom characteristics for training and testing data are depicted in Table 1. The proportions were consistent between the two data sets, with 17% and 20% of patients indicating the presence of AL in the training and testing data sets, respectively. Patients with AL were generally male, with ages ranging from 50 to 70 years, who were more likely to be smokers and heavy drinkers, and more likely to experience hypertension, peripheral vascular disease, and cardiac arrhythmia.

**Figure 2:**
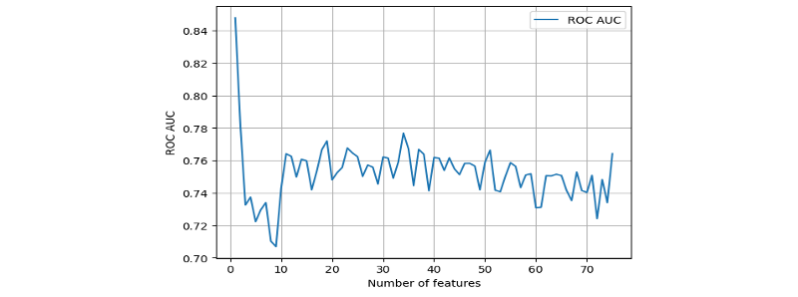
Change of area under the receiver operating characteristic by the number of features.

**Figure 3:**
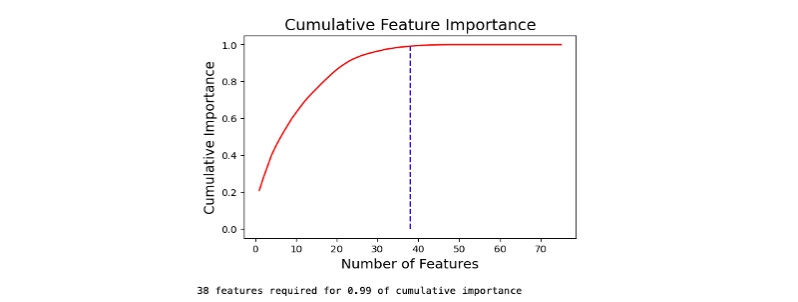
Number of features and cumulative feature importance.

Feature selection using the training data set provided us the independent risk factors panel predicting AL. The plot of change of AUROC over the number of features indicated 34 features would yield a relatively high AUROC value (AUROC=0.78; Figure 2). The cumulative importance score plot identified at least 38 features required for our final model (Figure 3; Table 2) [16,17].

**Table 1:** Demographic and symptom characteristics in the data sets defined by the presence or absence of AL.

		Training data set			Testing data set		
		No	Yes	*P* values^a^	No	Yes	*P* values
Presence of anastomotic leakage, n (%)		531 (83)	108 (17)		57 (80)	14 (20)	
**Age, n (%)**							
	<50	64 (12)	80 (15)	.36	58 (11)	37 (7)	.06
	50-59	196 (37)	207 (39)		271 (51)	112 (21)	
	60-69	218 (41)	191 (36)		170 (32)	303 (57)	
	>=70	53 (10)	53 (10)		37 (7)	74 (14)	
**Gender, n (%)**							
	Male	419 (79)	451 (85)	.17	409 (77)	377 (71)	.66
	Female	112 (21)	80 (15)		122 (23)	154 (29)	
**BMI, n (%)**							
	15-19	27 (5)	48 (9)	.74	37 (7)	37 (7)	.73
	20-25	340 (64)	287 (54)		372 (70)	340 (64)	
	>=26	165 (31)	196 (37)		122 (23)	154 (29)	
**Ever smoked, n (%)**							
	No	207 (39)	191 (36)	.53	234 (44)	228 (43)	.95
	Yes	324 (61)	340 (64)		297 (56)	303 (57)	
**Ever alcohol heavy drinker, n (%)**							
	No	212 (40)	159 (30)	.04	250 (47)	303 (57)	.52
	Yes	319 (60)	372 (70)		281 (53)	228 (43)	
**Presence of aortic calcification, n (%)**							
	No	419 (79)	292 (55)	<.001	457 (86)	303 (57)	.01
	Yes	112 (21)	239 (45)		74 (14)	228 (43)	
**Presence of celiac trunk calcification, n (%)**							
	No	473 (89)	366 (69)	<.001	457 (86)	457 (86)	.98
	Yes	58 (11)	165 (31)		74 (14)	74 (14)	
**FEV1% category, n (%)**							
	30-59	21 (4)	27 (5)	.13	37 (7)	74 (14)	<.001
	60-79	122 (23)	165 (31)		101 (19)	377 (71)	
	80-130	388 (73)	340 (64)		393 (74)	74 (14)	
**Abdominal surgery, n (%)**							
	No	473 (89)	446 (84)	.18	446 (84)	494 (93)	.41
	Yes	58 (11)	85 (16)		85 (16)	37 (7)	
**Presence of cardiac arrhythmia, n (%)**							
	No	462 (87)	441 (83)	.31	457 (86)	457 (86)	.98
	Yes	69 (13)	90 (17)		74 (14)	74 (14)	
**Presence of peripheral vascular disease, n (%)**							
	No	515 (97)	473 (89)	<.001	531 (100)	531 (100)	–
	Yes	16 (3)	58 (11)		0 (0)	0 (0)	
**Presence of hypertension, n (%)**							
	No	409 (77)	340 (64)	<.001	398 (75)	377 (71)	.76
	Yes	122 (23)	191 (36)		133 (25)	154 (29)	
**Ever used hypotension drug, n (%)**							
	No	457 (86)	425 (80)	.10	457 (86)	419 (79)	.50
	Yes	74 (14)	106 (20)		74 (14)	112 (21)	
**Ever taken insulin, n (%)**							
	No	504 (95)	489 (92)	.13	510 (96)	457 (86)	.12
	Yes	27 (5)	42 (8)		21 (4)	74 (14)	
**Lesion length category, n (%)**							
	1-3	117 (22)	74 (14)	.06	96 (18)	154 (29)	.25
	4-5	218 (41)	234 (44)		297 (56)	303 (57)	
	6-10	181 (34)	202 (38)		138 (26)	74 (14)	
	>10	16 (3)	27 (5)		0 (0)	0 (0)	
**Position of lesion, n (%)**							
	Upper esophagus	175 (33)	149 (28)	.90	186 (35)	303 (57)	.05
	Middle esophagus	212 (40)	234 (44)		186 (35)	191 (36)	
	Lower esophagus	69 (13)	96 (18)		74 (14)	37 (7)	
	Upper-middle esophagus	37 (7)	16 (3)		37 (7)	0 (0)	
	Lower-middle esophagus	27 (5)	37 (7)		27 (5)	0 (0)	
	Multi-position	16 (3)	5 (1)		21 (4)	0 (0)	
**ASA^b^ physical status classification, n (%)**							
	1	117 (22)	80 (15)	<.001	122 (23)	37 (7)	.04
	2	372 (70)	356 (67)		398 (75)	419 (79)	
	3	42 (8)	101 (19)		11 (2)	74 (14)	
**Blood transfusion, n (%)**							
	No	127 (24)	96 (18)	.97	196 (37)	154 (29)	.14
	Yes	404 (76)	435 (82)		335 (63)	377 (71)	
**Type of anastomotic, n (%)**							
	No	425 (80)	430 (81)	.79	435 (82)	340 (64)	.19
	Yes	106 (20)	101 (19)		96 (18)	191 (36)	
**Tube stomach, n (%)**							
	No	181 (34)	170 (32)	.07	101 (19)	191 (36)	.65
	Yes	350 (66)	361 (68)		430 (81)	340 (64)	
**Surgical approach, n (%)**							
	Nonthoraco-laparoscopy	181 (34)	133 (25)	.16	186 (35)	154 (29)	.68
	Thoraco-laparoscopy	350 (66)	398 (75)		345 (65)	377 (71)	
**Laparoscope, n (%)**							
	No	202 (38)	165 (31)	.03	234 (44)	266 (50)	.77
	Yes	329 (62)	366 (69)		297 (56)	266 (50)	
**Histology grade, n (%)**							
	0	276 (52)	218 (41)	.80	281 (53)	303 (57)	.95
	1	255 (48)	313 (59)		250 (47)	228 (43)	
**T classification, n (%)**							
	0	42 (8)	27 (5)	.14	37 (7)	37 (7)	.31
	1	53 (10)	58 (11)		48 (9)	74 (14)	
	2	281 (53)	303 (57)		281 (53)	228 (43)	
	3	159 (30)	143 (27)		170 (32)	191 (36)	
**Multiple primary, n (%)**							
	No	499 (94)	478 (90)	.37	494 (93)	531 (100)	.36
	Yes	32 (6)	53 (10)		37 (7)	0 (0)	
**N classification, n (%)**							
	1	133 (25)	122 (23)	.26	175 (33)	154 (29)	.62
	2	101 (19)	101 (19)		64 (12)	37 (7)	
	3	239 (45)	234 (44)		212 (40)	191 (36)	
	4	32 (6)	42 (8)		58 (11)	112 (21)	
	5	27 (5)	32 (6)		21 (4)	37 (7)	
**Thyroglobulin level, n (%)**							
	<1.7	441 (83)	473 (89)	.15	473 (89)	457 (86)	.70
	>=1.7	90 (17)	58 (11)		58 (11)	74 (14)	
**Tumor vascular permeability, n (%)**							
	<20	372 (70)	372 (70)	.92	324 (61)	419 (79)	.23
	>=20	159 (30)	159 (30)		207 (39)	112 (21)	
**Postoperative ventilator-assisted breathing, n (%)**							
	No	515 (97)	473 (89)	<.001	520 (98)	457 (86)	.04
	Yes	16 (3)	58 (11)		11 (2)	74 (14)	
**Lung infection, n (%)**							
	No	510 (96)	478 (90)	<.001	494 (93)	494 (93)	0.99
	Yes	21 (4)	53 (10)		37 (7)	37 (7)	
**Pleural effusion or empyema, n (%)**							
	No	515 (97)	446 (84)	<.001	520 (98)	419 (79)	<.001
	Yes	16 (3)	85 (16)		11 (2)	112 (21)	
**Incision fat liquefaction and infection, n (%)**							
	No	494 (93)	398 (75)	<.001	467 (88)	494 (93)	.59
	Yes	37 (7)	133 (25)		64 (12)	37 (7)	
Hospital Length of Stay, mean (SD)		13.08 (7.2)	35.56 (23.2)	<.001	12.75 (4.13)	29.36 (16.72)	<.001

^a^*P* values are reported using the Pearson chi-square method.

^b^The American Society of Anesthesiologists Physical Status Classification System is a well-established assignment that assesses and communicates a patient’s pre-anesthesia medical comorbidities.

**Table 2:** Comparison of the model’s performance metrics with different machine learning classifiers.

	Logistic regression	Support vector classifier	Decision tree	Random forest	Gaussian naïve Bayes	Best score
Accuracy	0.91	0.90	0.84	0.90	0.82	Logistic regression
Precision	0.81	0.81	0.55	0.80	0.50	Logistic regression
Recall (sensitivity)	0.64	0.59	0.58	0.59	0.68	Gaussian naïve Bayes
F1 score	0.71	0.68	0.56	0.67	0.58	Logistic regression
AUROC	0.76	0.72	0.65	0.70	0.67	Logistic regression

After removing the features with only one unique value and one strong collinear feature, we identified the 36 most important risk factors to create our model, ensuring robustness and stability. Our panel of predictive risk features, listed and ranked by their importance, is shown in Table 3. The preoperative factors included patient’s age, gender, BMI, smoking and alcohol intake, malnutrition status, ASA index, cardiovascular disease (aortic calcification, celiac trunk calcification, peripheral vascular disease, cardiac arrhythmia, and hypertension), obstructive lung diseases test scores (forced vital capacity ratio [FEV1%], transfer factor for carbon monoxide [TLco]), surgical history (abdominal surgery), drug usage (insulin and hypertension drugs), and cancer staging (TNM classification of malignant tumors). The intra-operative factors included operation time, lesion length and position of the lesion, availability of blood transfusion, and surgical approaches. The postoperative factors contained a prolonged hospital stay length and surgical complications such as arrhythmia, lung infection, pleural effusion, and fat liquefaction of incisions.

**Figure 4:**
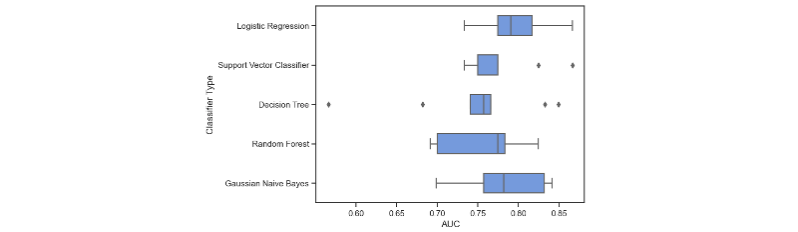
Comparison of model’s area under the receiver operating characteristic with different machine learning classifiers.

After the feature panel was determined, the model selection metrics showed the logistic regression with least absolute shrinkage and selection operator (LASSO) obtained the best median AUROC score, making it the most reliable ML classifier for this data set (Figure 4). In addition, it is more easily interpreted by the medical audience. To further improve the model’s performance and overcome the potential overfitting risk caused by the large number of features, we added penalty items into the logistic model and used a grid search to find the optimal type of penalty (LASSO) and the hyperparameters used in the penalty term. The model’s performance was substantially improved using LASSO regularization.

Based on the final prediction model, multivariate logistic regression recognized the most significant risk factors as follows: aortic calcification (OR 2.77, 95% CI 1.32-5.81), celiac trunk calcification (OR 2.79, 95% CI 1.20-6.48) , FEV1% (OR 0.51, 95% CI 0.30-0.89); TLco (OR 0.56, 95% CI 0.27-1.18), peripheral vascular disease (OR 4.97, 95% CI 1.44-.07), laparoscope (OR 3.92, 95% CI 1.23-12.51), postoperative hospital length of stay (OR 1.17, 95% CI 1.13-1.21), vascular permeability activity (OR 0.46, 95% CI 0.14-1.48), and fat liquefaction of incisions (OR 4.36, 95% CI 1.86-10.21).

**Figure 5:**
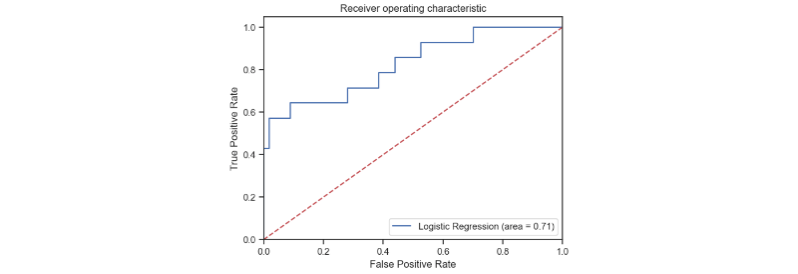
Final model performance presented by the area under the receiver operating characteristic.

We used the testing data set to validate the model’s predictive ability. The AUROC curve was used to evaluate the model fitting. The logistic regression model with LASSO resulted in a clinically relevant AUROC of 71%, indicating good model performance (Figure 5). We also presented the AUROC, accuracy, recall, precision, and F1 score as extended metrics of both data sets. The AUROC accuracy and precision were consistent between the 2 data sets (Table 4).

**Table 3:** Panel of prediction factors selected in the training data set.

Features		Importance	Remain in model after correlation-based feature selection	Regression coefficients in final model to predict AL	*P* values in final model to predict AL	Odds ratio for AL (95% CI)
**Preoperative factors**						
	Aortic calcification	29.8	Yes	1.0203	0.0069	2.77(1.32-5.81)
	Celiac trunk calcification	32.4	Yes	1.0275	0.0167	2.79(1.20, 6.48)
	Forced vital capacity ratio (FEV1%)	48.5	Yes	-0.6653	0.0177	0.51(0.30-0.89)
	Transfer factor for carbon monoxide (TLCO) by single-breath (SB) method (%)	51.8	Yes	-0.5820	0.0111	0.56(0.27-1.18)
	Peripheral vascular disease	17.6	Yes	1.6026	0.0109	4.97(1.44-17.07)
**Intra-operative and postoperative factors**						
	Laparoscope	40.3	Yes	1.3665	0.0209	3.92(1.23-12.51)
	Postoperative hospital stay	219.6	Yes	0.1571	0.0000	1.17(1.13-1.21)
	Tumor vascular permeability (laboratory test)	20.1	Yes	-0.7663	0.0507	0.46(0.14-1.48)
	Incision fat liquefaction and infection	23.5	Yes	1.4718	0.0007	4.36(1.86-10.21)

**Table 4:** Metrics for evaluating the machine learning application.

	Accuracy	Precision	Recall	F1 score	AUROC
Training data set	87%	88%	98%	93%	72%
Testing data set	87%	86%	43%	57%	71%

## Discussion

### Principal Findings

The study provided a panel with 36 features for predicting AL in patients who undergo esophagectomies, including detailed symptoms, surgical technical details, and complications. It can identify the presence of AL with high accuracy (87%) and precision (88%). In addition, our panel of risk factors is supported by the previous randomized controlled trials (RCTs), retrospective cohort studies, and meta-analyses [18-21].

Gaining insight into the risk factors of AL is crucial for designing an evidence-based treatment algorithm that will help guide clinical teams to perform timely AL management and support preoperative and postoperative optimization. Among the most important risk factors for AL development are 4 preoperative comorbidities, laparoscopic esophagectomies, and some postoperative complications. In general, the 4 preoperative comorbidities and most postoperative complications are modifiable factors that may guide patient-centered strategies. Full awareness of preoperative risk factors is essential for identifying high-risk patients and appropriately targeting them to mitigate the severe clinical consequences of AL. For example, if the patient is noticeably concerned about the postoperative AL complications, the clinical team might consider other nonsurgery treatments, such as chemotherapy. Likewise, the postoperative conditions help clinicians actively monitor patients’ recovery after esophagectomies, allowing them to identify AL early.

### Preoperative Risk Factors (Patient Screening and Preparation)

The 4 preoperative comorbidities significantly linked to increased AL risk are peripheral vascular disease, aortic calcification, celiac trunk calcification, and COPD indicators (FEV1% and TLco results). In addition, hypertension, diabetes mellitus, and coronary artery disease are independent risk factors. These preoperative comorbidities all have a negative impact on microvascular perfusion, and corresponding atherosclerosis might affect the etiology of AL [22]. Older esophageal cancer patients, whose nutrition status is often impaired, have a higher rate of atherosclerosis and new-onset atrial fibrillation, making them vulnerable to AL. Moreover, there might be an association between supra-aortic and coronary atherosclerosis and AL, implying that general atherosclerosis scores could predict AL risk. To optimize the preoperative screening of esophageal cancer patients, our study suggests a thorough investigation of atherosclerosis-related risks factors and continuous monitoring of perioperative hemodynamics is essential to prevent AL.

### Intra-operative Risk Factors (Surgical-Technical Aspects)

The prediction model indicates a laparoscopic esophagectomy alone increases the odds of AL by 3.92. However, the results require cautious interpretation. While esophagectomy surgical technique has evolved considerably, the scientific evidence regarding the superiority of specific esophagectomy techniques in reducing morbidity, such as AL, is not robust [23]. Our data collection started when the MIE was just initiated in our cancer center. The majority of our thoracic surgeons exclusively performed open esophagectomies, and they were at the early stages of learning MIE. The increased odds of AL linked to laparoscopic esophagectomies were primarily explained by the proficiency gain curve–associated morbidity since laparoscopic esophagectomies require extensive and adequate training for our thoracic surgeons.

### Postoperative Risk Factors (Medical Outcomes)

Accurate monitoring of postoperative complications has a significant impact on AL development [24]. The adverse medical outcomes associated with AL generally found in this study are the occurrence of fat liquefaction of incisions, reduced vascular permeability, and prolonged lengths of hospital stays. Recent studies support the impact of our prediction on the outcomes. Kamarajah et al [18] summarized the meta-analysis results from previous studies in this field and confirmed the importance of the pulmonary complications (OR 4.54, 95% CI 2.99-6.89), cardiac complications (OR 2.44, 95% CI 1.77-3.37), and prolonged hospital stays (OR 5.91, 95% CI 1.41-24.97) [18]. Postoperative management should pay attention to any possible incision infections during follow-ups to prevent further development of anastomotic stricture.

### Comparison With Prior Work

One advantage of this study is the effective feature selection. Conventional statistical analyses identified various inconsistent risk factors which were cross-correlated. We approached this challenge by combining univariate and multivariable feature selection techniques to produce a stable panel. Important features were selected, internally cross-validated, and not connected to a specific learning algorithm; therefore, minimal human bias was involved [15].

### Limitations

This study had some limitations. First, because this is a retrospective study that includes consecutive patients, we could not determine the long-term sequelae of AL in the current database, specifically pathological development. Second, the study was limited to a single center, and the results are therefore representative for the specific geographic region and cannot be generalized. Before extrapolating the model to other facilities, it is necessary to consider other risk factors such as geographical and treatment background. However, our hospital is one of China's top cancer research centers and can collect sufficient surgical esophageal cancer cases. Third, due to the complexity of ML models, substantial computing power is required for practical deployment. However, benefiting from the current development of electronic medical records and embedding automated ML algorithms can enable efficient and expedient risk calculations and substantially improve the convenience of utilizing ML models.

### Conclusions

The ML prediction model of AL provides insight into the important risk factors for designing evidence-based clinical management that will help guide physicians regarding AL prevention and treatment. However, additional prospective data collection is needed using a cohort study design or RCT design in multiple medical settings to confirm our findings' validity and establish a better risk prediction model. 

## Appendix

Multimedia Appendix 1 Risk Factors Panel.

Multimedia Appendix 2 TRIPOD Checklist.

## Abbreviations

AL: anastomotic leakage
ASA: The American Society of Anesthesiologists
AUROC: area under the receiver operating characteristic curve
COPD: chronic obstructive pulmonary disease
FEV: forced expiratory volume
GBM: gradient boosting model
LASSO: least absolute shrinkage and selection operator
ML: machine learning
MIE: minimally invasive esophagectomy
OR: odds ratio
RCT: randomized controlled trials
TLco: transfer factor for carbon monoxide
TRIPOD: Transparent Reporting of a Multivariable Prediction Model for Individual Prognosis or Diagnosis
