# Supplementary material for: Prediction Model of Anastomotic Leakage Among Esophageal Cancer Patients After Receiving an Esophagectomy: Machine Learning Approach
Source: JMIR Med Inform. 2021 Jul 27;9(7):e27110. doi: 10.2196/27110 (PMC8367102; doi:10.2196/27110)
Supplement: Multimedia Appendix 1 [file medinform_v9i7e27110_app1.docx]

Appendix A Risk Factors Panel Identified from the ML model

| Features | Importance | Remain in model after correlation-based feature selection | Regression coefficients in final model to predict AL | P values in final model to predict AL |
| --- | --- | --- | --- | --- |
| Age | 68.7 | Yes | -0.2725 | 0.1712 |
| Gender | 7.5 | Yes | -0.3930 | 0.4272 |
| BMI | 33.5 | Yes | 0.1022 | 0.7319 |
| Smoking history | 39.1 | Yes | -0.8423 | 0.0457 |
| Alcohol history | 17.1 | Yes | 0.9168 | 0.0629 |
| Aortic calcification | 29.8 | Yes | 1.0203 | 0.0069 |
| Celiac Trunk Calcification | 32.4 | Yes | 1.0275 | 0.0167 |
| Forced vital capacity ratio (FEV1%) | 48.5 | Yes | -0.6653 | 0.0177 |
| Transfer factor for carbon monoxide (TLCO) by single-breath (SB) method (%) | 51.8 | Yes | -0.5820 | 0.0111 |
| Peripheral vascular disease | 17.6 | Yes | 1.6026 | 0.0109 |
| Abdominal surgery | 7 | Yes | 0.6908 | 0.1065 |
| Cardiac arrhythmia | 16.8 | Yes | -0.4787 | 0.3417 |
| Hypertension | 23.6 | Yes | -0.8591 | 0.1074 |
| Taking hypertension drug | 9.9 | Yes | 0.5573 | 0.3936 |
| Taking insulin | 5 | Yes | 0.6235 | 0.3303 |
| Lesion length | 59.2 | Yes | 0.2533 | 0.2583 |
| Position of lesion | 38.3 | Yes | -0.2124 | 0.1365 |
| The American Society of Anesthesiologists (ASA) physical status classification | 31.3 | Yes | -0.2575 | 0.3998 |
| Operation time | 11.5 | Yes | -0.5768 | 0.1287 |
| Blood transfusion | 4.7 | Yes | 0.1312 | 0.7706 |
| Type of anastomotic | 17 | Yes | 0.2104 | 0.5834 |
| Tube stomach | 15.9 | Yes | 0.2082 | 0.5991 |
| Surgical approach | 13.1 | Yes | -0.7736 | 0.1923 |
| Laparoscope | 40.3 | Yes | 1.3665 | 0.0209 |
| Postoperative hospital stay | 219.6 | Yes | 0.1571 | 0.0000 |
| Tumor Vascular Permeability (Laboratory Test) | 20.1 | Yes | -0.7663 | 0.0507 |
| Incision fat liquefaction and infection | 23.5 | Yes | 1.4718 | 0.0007 |
| Histology Grade | 26.7 | Yes | 0.0526 | 0.8045 |
| Multiple primary | 5.1 | Yes | 0.9873 | 0.1119 |
| T Classification | 43.9 | Yes | -0.1342 | 0.4195 |
| N Classification | 42.1 | Yes | 0.0872 | 0.6688 |
| Thyroglobulin Level | 4.3 | Yes | -0.9206 | 0.0976 |
| Postoperative ventilator-assisted breathing | 8.3 | Yes | 1.0762 | 0.1018 |
| Lung infection | 9.1 | Yes | 0.1466 | 0.8385 |
| Pleural effusion or empyema | 6.5 | Yes | 0.9949 | 0.1446 |
